# Diverse retinal-kidney phenotypes associated with *NPHP1* homozygous whole-gene deletions in patients with kidney failure

**DOI:** 10.1007/s44162-024-00031-4

**Published:** 2024-03-01

**Authors:** Gavin Esson, Ian Logan, Katrina Wood, Andrew C. Browning, John A. Sayer

**Affiliations:** 1 Renal Services, The Newcastle Upon Tyne Hospitals NHS Foundation Trust, Newcastle Upon Tyne, UK; 2 Histopathology Department, The Newcastle Upon Tyne Hospitals NHS Foundation Trust, Newcastle Upon Tyne, NE1 4LP UK; 3 Ophthalmology Department, The Newcastle Upon Tyne Hospitals NHS Foundation Trust, Newcastle Upon Tyne, NE1 4LP UK; 4 Biosciences Institute, Faculty of Medical Sciences, Newcastle University, Central Parkway, Newcastle Upon Tyne, NE1 3BZ UK; 5 NIHR Newcastle Biomedical Research Centre, Newcastle Upon Tyne, UK

**Keywords:** Kidney failure, NPHP1 homozygous deletion, Nephronophthisis, Disease, Senior-Løken syndrome, Retinal dystrophy

## Abstract

A precise diagnosis in medicine allows appropriate disease-specific management. Kidney failure of unknown aetiology remains a frequent diagnostic label within the haemodialysis unit and kidney transplant clinic, accounting for 15–20% of these patients. Approximately 10% of such cases may have an underlying monogenic cause of kidney failure. Modern genetic approaches can provide a precise diagnosis for patients and their families. A search for extra-renal disease manifestations is also important as this may point to a specific genetic diagnosis. Here, we present two patients where molecular genetic testing was performed because of kidney failure of unknown aetiology and associated retinal phenotypes. The first patient reached kidney failure at 16 years of age but only presented with a retinal phenotype at 59 years of age and was found to have evidence of rod-cone dystrophy. The second patient presented with childhood kidney failure at the age of 15 years and developed visual difficulties and photophobia at the age of 32 years and was diagnosed with cone dystrophy. In both cases, genetic tests were performed which revealed a homozygous whole-gene deletion of *NPHP1*-encoding nephrocystin-1, providing the unifying diagnosis of Senior-Løken syndrome type 1. We conclude that reviewing kidney and extra-renal phenotypes together with targeted genetic testing was informative in these cases of kidney failure of unknown aetiology and associated retinal phenotypes. The involvement of an interdisciplinary team is advisable when managing such patients and allows referral to other relevant specialities. The long time lag and lack of diagnostic clarity and clinical evaluation in our cases should encourage genetic investigations for every young patient with unexplained kidney failure. For these and similar patients, a more timely genetic diagnosis would allow for improved management, a risk assessment of kidney disease in relatives, and the earlier identification of extra-renal disease manifestations.

**Supplementary Information:**

The online version contains supplementary material available at 10.1007/s44162-024-00031-4.

## Introduction

Many patients across the spectrum of general medicine will have a clear diagnosis with a well-established treatment; however, most clinicians will be familiar with diagnostic uncertainty. Patients without a precise diagnosis often describe experiencing the “diagnostic odyssey” with years of inappropriate treatments, investigations, and onward referrals [1, 2]. This is costly and time-consuming for healthcare providers and takes a large mental and physical toll on patients [3].

Patients with kidney failure of unknown aetiology provide an excellent example of a group of undiagnosed patients. In registry data from kidney units, this group of patients represent 10–20% of all adult kidney failure cases [4–6]. A diagnostic kidney biopsy in this patient group is often not performed due to atrophic small kidneys which are at high risk of bleeding, or if performed can lead to non-specific findings observed in late stages of chronic kidney disease (CKD). In patients with kidney failure of unknown aetiology, an exact diagnosis can be important for considerations relating to kidney transplantation. The primary or original kidney disease may affect transplant graft survival by disease recurrence or rejection, and living-related kidney donation may be inadvisable where family members may be at risk of the same disease.

The estimated prevalence of CKD on unknown cause is around 10–20% in adult patients [7–9], and therefore, genetic testing will enable a proportion of such patients to have a definitive diagnosis. Indeed, the diagnostic yield of modern day sequencing approaches is 30% for childhood cases and between 6 and 30% for adult cases. Recently, guidelines to help the nephrologist navigate genetic testing modalities and their indications have been published [10, 11]. Other benefits of a precise diagnosis include directed screening for systemic and extra-renal associations, identifying potential specific therapies, empowering the patient with a diagnosis, and allowing families to consider the implications for offspring and relatives [12]. For a growing number of patients and their monogenic disorders, preimplantation genetic testing is now possible, which allows prospective parents to make informed choices about and to avoid passing on the heritable disease, which include a range of monogenic kidney conditions, to their offspring [13, 14]. The ability to provide a diagnosis of the aetiology of kidney failure for the patient and screen at-risk family members is becoming increasingly feasible given the recent advancements in genetic diagnostics through next-generation sequencing (NGS) approaches such as whole-genome sequencing (WGS) that are becoming embedded into modern healthcare systems [15]. Utilising these resources should allow a more comprehensive genetic testing for patients with CKD and kidney failure [16, 17].

The NHS has acknowledged these technological advances and their importance to patient care and in 2022 launched the “Rare Disease Action Plan”—aimed at increasing diagnosis, knowledge, and support for rare diseases [18]. For clinicians and patients faced with diagnostic uncertainty, there has never been a better time to re-investigate with new diagnostic strategies including NGS genetic testing.

Here, we provide examples of two such patients in whom a precise genetic diagnosis was obtained decades after the initial presentation of kidney failure, allowing a unifying diagnosis of kidney and extra-renal disease manifestations.

### Patient 1

A 13-year-old male with no previous medical history was referred to our Renal Services Department, in 1969, with a 1-year history of lethargy, increased thirst and reduced kidney function. Abdominal ultrasound scanning showed normal-sized kidneys with some bilateral small medullary cysts. He underwent a kidney biopsy which revealed chronic damage with tubulointerstitial nephritis. A tentative diagnostic label of medullary cystic kidney disease was given. This was treated with supportive measures. By the age of 16 years, he had evidence of progressive chronic kidney disease resulting in kidney failure, and he required treatment with haemodialysis. There was no other known family history of kidney disease, he had a healthy younger brother, and both parents were fit and well with normal kidneys on ultrasound examination.

**Fig. 1:**
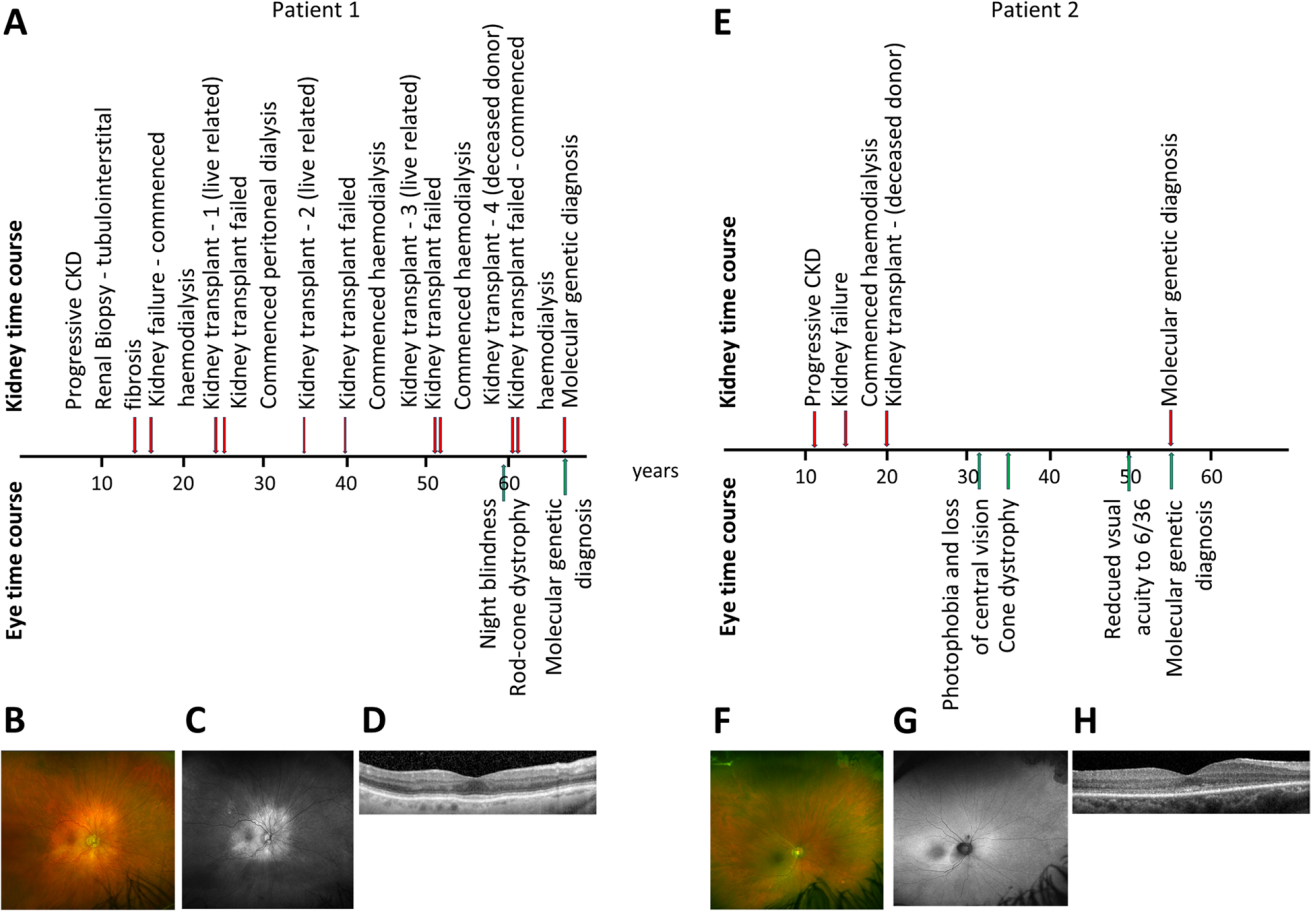
Clinical time course and retinal phenotypes of two cases of Senior-Løken syndrome. **A** Time course of patient 1 showing presentations of kidney and eye features. **B** Wide-angle colour fundus images of patient 1 showing a normal retinal appearance apart from some peripapillary pallor. **C** Wide-angle autofluorescence images of patient 1 showing reduced signal outside of the peripapillary area indicating widespread loss of retinal pigment epithelium/photoreceptor cells. **D** Posterior pole macular optical coherence tomography (OCT) images of patient 1 show well-preserved photoreceptor integrity. **E** Time course of patient 2 showing presentations of kidney and eye features. **F** Wide-angle colour fundus images of patient 2 showing a normal retinal appearance. **G** Wide-angle autofluorescence images of patient 2 showing a normal appearance apart from some far peripheral paving stone retinal pigment epithelium degeneration. **H** Posterior pole macular OCT images showing outer photoreceptor granularity indicating cone disruption

At the age of 24, the patient received a living-related kidney transplant from his mother, which lasted just 18 months before failing and was surgically removed. He then received treatment with peritoneal dialysis for around 10 years. The patient then received a second living-related kidney transplant from his father, which was functional for around 5 years. Following this, he had a third living-related kidney transplant from his brother, which failed immediately due to acute graft rejection. Around 10 years later, he received a fourth kidney transplant from an unknown deceased donor, which also failed with acute graft rejection and biopsy-proven thrombotic microangiopathy which was likely to represent antibody-mediated rejection. He is now maintained on in-centre haemodialysis via a fistula (Fig. 1A).

In terms of extra-renal phenotypes, the patient was initially seen in the ophthalmology department at the age of 59 when he complained of difficulty seeing in the dark (Fig. 1A). His visual acuity was 6/9 bilaterally, and his retinal examination and wide-angle imaging were unremarkable except for some loss of retinal pigment around his optic discs with an associated increase in localised fundus autofluorescence signal (Fig. 1B, C). A macular optical coherence tomography (OCT) scan showed an intact ellipsoid zone layer indicating well-preserved macula photoreceptor structure (Fig. 1D). An International Society for Clinical Electrophysiology of Vision (ISCEV) standard full-field electroretinogram (ERG) revealed widespread retinal photoreceptor dysfunction in a pattern consistent with a rod-cone dystrophy (Additional file 1: Fig. S1). Brain MRI did not reveal any cerebellar vermis hypoplasia, and plain X-rays of the chest and long bones also showed no abnormalities. There was no clinical evidence of oculomotor apraxia.

Given the poor kidney transplant outcomes and the early-onset nature of his kidney failure and his developing retinal phenotype, his case was reviewed to determine if a precise diagnosis was possible. His early-onset progressive CKD was suggestive of a monogenic cause, although there was no family history of kidney disease, diabetes, gout, deafness or haematuria. Following informed consent, the patient underwent genetic testing. A 15-gene, targeted NGS gene panel was undertaken (R202, tubulointerstitial kidney disease, Additional file 1: Table S1) and revealed a homozygous whole-gene deletion of *NPHP1*, consistent with a diagnosis of nephronophthisis type 1 and a unifying diagnosis of Senior-Løken syndrome type 1. A microarray revealed the homozygous deletion to be 127 kb in size and included exon 1 of the flanking *MALL* gene, the typical finding of *NPHP1* whole-gene deletions. In addition, a retinal NGS panel was also performed (R32 Retinal disorders, containing 412 genes, Additional file 1: Table S2) and identified a heterozygous variant in *ABCC6*, p.(Arg1141X) (Additional file 1: Table S3). The ACMG criteria suggest this nonsense allele is pathogenic.

### Patient 2

This female patient had presented at 12 years of age with anaemia, malaise and a progressive decline in kidney function resulting in kidney failure by 15 years of age. Kidney imaging had shown bilateral small kidneys which were not biopsied. She received a kidney transplant at the age of 20 years and has maintained excellent transplant function since then. The patient is currently 54 years of age (Fig. 1E). She has no siblings or children.

The patient started to develop difficulty with her central vision and photophobia at the age of 32 years (Fig. 1E). Her visual acuity at this point was 6/9 right and 6/12 left, with an unremarkable ocular examination. At the age of 35 years, retinal electrophysiology revealed minimal cone function but normal rod function, consistent with a diagnosis of cone dystrophy (Additional file 1: Fig. S1). By the age of 50 years, her acuity had declined to 6/36 bilaterally. At this stage, retinal examination and wide-angle imaging were unremarkable except for some peripheral paving stone degeneration which can be a normal finding (Fig. 1F). Wide-angle fundus autofluorescence imaging was normal (Fig. 1G). A macular OCT scan showed widespread fragmentation and loss of the ellipsoid zone layer indicating loss of macula photoreceptor structure (Fig. 1H). Genetic testing was initiated to elucidate the underlying cause of the retinal-kidney phenotype. A retinal NGS panel (R32 retinal disorders, Additional file 1: Table S2) revealed a homozygous whole-gene deletion of *NPHP1* which would account for both the retinal phenotype and her early-onset kidney failure and give a unifying diagnosis of Senior-Løken syndrome type 1. A microarray confirmed the homozygous deletion to be 127 kb in size and included exon 1 of the flanking *MALL* gene, again typical of reported *NPHP1* whole-gene deletions. The R32 retinal disorder gene panel also revealed that the patient was also heterozygous for a rare variant in *USH1C*, p.(Arg196X) (Additional file 1: Table S3). The ACMG criteria suggest this nonsense allele is pathogenic. No additional extra-renal manifestations were identified with normal brain MRI and normal plain X-rays of the chest and long bones. There was no also clinical evidence of oculomotor apraxia.

## Discussion

Inherited causes of kidney failure are numerous, and associated kidney phenotypes include cystic kidney diseases, glomerular diseases and tubulointerstitial diseases. Autosomal dominant polycystic kidney disease (ADPKD) accounts for around 10% of all adult kidney failure, and a precise molecular genetic diagnosis helps in the understanding of the underlying cause and suitability for future clinical trials and helps identification of extra-renal manifestations. The cystic kidney disease phenotype may be variable, and new genes explaining atypical cases of ADPKD are being identified [19, 20]. Other monogenic causes of kidney failure may also have variable clinical features and can be more difficult to diagnose both clinically and genetically. Other inherited heterogeneous disorders include Alport syndrome, familial focal segmental glomerulosclerosis (FSGS), autosomal dominant tubulointerstitial kidney disease (ADTKD) and nephronophthisis [11]. Here, a kidney biopsy may help to define a probable diagnosis, but there is often phenotypic overlap between genetic disorders allowing for diagnostic confusion. For example, patients with *COL4A4* pathogenic variants were recently described to be the most frequent cause of FSGS [21], and ADTKD can be clinically and histologically indistinguishable from nephronophthisis [22, 23]. A positive family history may help in determining the probable pattern of inheritance, but often in recessively inherited diseases or in de novo cases of dominant disease, there may not be any suggestion of a familial pattern of disease.

Nephronophthisis (NPHP) is an autosomal recessive kidney disease, which is typically paediatric in onset [24]. It is the most common genetic cause of kidney failure in children with a median onset of kidney failure of 13 years [25, 26]. More recently, it has been shown that NPHP may lead to adult-onset kidney failure [27, 28]. There may be very few clinical clues that point towards a diagnosis of NPHP. Typically, there may be polyuria and polydipsia and sometimes secondary enuresis [29]. A kidney ultrasound scan may show loss of corticomedullary differentiation and sometimes small medullary cysts [30]. The urine is typically bland, and there may not be severe hypertension. A kidney biopsy may show interstitial fibrosis, tortuous and atrophic tubules and segmented tubular basement membrane thickening [31]. There may be cystic dilatation of the tubules. Much of these findings are non-specific, and in cases of kidney failure of unknown cause, a histopathological diagnosis should be superseded by a molecular genetic diagnostic approach, because genetic screening allows for early diagnosis and prevents the need for a kidney biopsy.

A recent study in a kidney failure population was informative. In this adult cohort of over 5000 Europeans, 0.5% of the cohort showed homozygous *NPHP1* deletions [32]. Interestingly, only 3 (12%) of this group of 26 patients were clinically diagnosed correctly, and the rest were labelled as CKD of unknown cause. Therefore, *NPHP1* whole-gene deletions in this cohort were a relatively frequent cause of adult-onset kidney failure.

Nephronophthisis, unlike some forms of glomerulonephritis, does not recur in the transplanted kidney, but the clinical course of recurrent failure of kidney transplants in patient 1 is noteworthy. The reason for repeated transplant failure is likely to be multifactorial and includes documented tacrolimus toxicity, acute tubular necrosis episodes, BK virus nephropathy and chronic rejection. The fourth graft showed biopsy evidence of likely antibody-mediated rejection, and it is likely that patient 1 had become highly sensitised by this stage with high levels of anti-HLA antibodies. In contrast, patient 2 enjoyed a long-lasting kidney transplant which did not show any rejection episodes.

There are several additional extra-renal manifestations that may be associated with NPHP which include retinal dystrophy, oculomotor apraxia, cerebellar vermis aplasia, liver fibrosis and skeletal dysplasia. The association of NPHP with retinal dystrophy is known as Senior-Løken syndrome [33, 34]. Visual impairment has been reported in approximately 20% of patients with NPHP caused by mutations in *NPHP1*, with findings ranging from mild retinal changes to early-onset blindness caused by retinitis pigmentosa or Leber’s congenital amaurosis [35]. A recent study that performed extensive ocular phenotyping of patients with homozygous deletions of *NPHP1* found visual impairment was usually mild with a mean visual acuity of 6/6 (range 6/6 to 6/24). In the majority of patients, wide-angle retinal imaging and central retinal fundus autofluorescence imaging were both normal, and the only significant retinal findings were macular granularity and outer macular photoreceptor abnormalities on OCT imaging [36]. Further phenotyping of this cohort using retinal electrophysiology demonstrated either normal retinal function or dysfunction in the pattern of a cone or cone/rod dystrophy; however, one patient (age 28 years) had severe retinal degeneration and an almost extinguished ERG. Interestingly, the patient was also found to be a carrier for a pathogenic variant in *CEP290* (another multi-system ciliopathy-related gene [37, 38]). These authors and others suggested that being a carrier for a disease-causing variant in another ciliopathy-related gene may modify the severity of the disease phenotype. This disease-modifying phenomenon may account for the different ocular phenotypes of our two cases. Despite both having identical homozygous deletions of *NPHP1* that extended into the 1st exon of *MALL*, case 1 was found to have a pattern of severe retinal dysfunction in keeping with a rod/cone dystrophy but maintained good visual acuity during follow-up, while case 2 demonstrated a gradual progression of visual loss over almost 20 years of follow-up and demonstrated a cone dystrophy pattern of retinal dysfunction and loss of macular outer photoreceptor structure in keeping with previous reports of this mutation [36]. In patient 1, we also identified a heterozygous rare nonsense variant in *ABCC6* which is predicted by ACMG criteria to be pathogenic (Additional file 1: Table S3). Biallelic variants in *ABCC6* cause pseudoxanthoma elasticum, a connective tissue disorder that can effect both the retina, typically leading to breaks in Bruch’s membrane of the retina (angioid streaks), and the kidney, resulting in calcification of the blood vessels. Interestingly, mild ocular phenotypes have been reported in heterozygous carriers [39], but such findings were not observed in patient 1 or any of his close relatives. Unfortunately, segregation studies concerning the *ABCC6* allele were unable to be carried out. The role of this additional allele is therefore uncertain.

Patient 2 was found to be a carrier of a rare, predicted pathogenic (according to ACMG criteria) nonsense allele in *USH1C* (Additional file 1: Table S3), a ciliopathy-related gene where biallelic variants are associated with type 1 Usher’s syndrome and autosomal recessive deafness. Interestingly, the *USCH1*-encoded protein harmonin is expressed in the synaptic terminals of both retinal photoreceptors and inner ear hair cells [40]. Usher syndrome type 1 is associated with early retinitis pigmentosa (usually evident within the first decade) [41]. Again, heterozygous carriers have been reported to have mild retinal phenotypes [42], and the impact of this allele on the overall retinal phenotype in patient 2 is unknown. No other retinal disease was seen in first-degree relatives, but in this family, segregation studies were unable to be carried out. Parental DNA is not available, and patient 2 has no siblings or children. It would be interesting to study more cases of genetically solved Senior-Løken syndrome to determine whether harbouring these additional heterozygous alleles modifies the eye phenotype of this condition.

Once a genetic diagnosis has been identified, there may be treatment implications depending on the specific underlying cause of kidney failure [43]. NPHP presently does not have specific treatments for its kidney or retinal manifestations, but ongoing research, facilitated by the identification of clinical cases, may yield future treatments. Indeed, retinal treatments for Leber congenital amaurosis secondary to *CEP290* disease-causing variants are being explored using intravitreal injection of either replacement gene supplements or CRISPR-based gene editing. Nevertheless, there are examples of monogenic kidney disorders where a precise diagnosis may dramatically affect patient management [44]. For example, primary hyperoxaluria type 1 will recur in a kidney transplant as the primary defect lies in the liver [45]. Patients with steroid-resistant nephrotic syndrome (SRNS) secondary to pathogenic variants in *COQ2*, *COQ6*, or *ADCK4* may respond to the administration of coenzyme Q_10_, which provides a valuable treatment option [46–49]. In addition, it may influence decisions regarding living-related organ donation and who is able to donate a kidney. This is very relevant in autosomal dominant forms of inherited kidney disease such as ADPKD secondary to *PKD1* and *PKD*2 disease-causing variants, and ADTKD secondary to *MUC1*, *UMOD*, *HNF1B*, and *REN* disease-causing variants [50]. Molecular diagnosis may influence choices regarding family planning and conception, especially in consanguineous families [51]. A precise molecular genetic diagnosis is vital information that can be used when couples and prospective parents are considering preimplantation genetic testing, which would allow them the choice to prevent passing on disease-causing variants to their offspring.

## Conclusion

These two case presentations demonstrate the value of revisiting a precise diagnosis in patients with kidney failure of unknown aetiology, especially in the context of extra-renal features such as retinal dystrophy. Modern-day genetic testing revealed the underlying condition and provided unifying diagnoses in both cases. This culture of defining precise causes of kidney failure will hopefully allow for individualised pre- and post-kidney transplant patient management and the assessment of risks of kidney disease and extra-renal manifestations in relatives. For these reasons, we advocate the consideration of genetic testing in all patients with kidney failure of unknown cause.

### Supplementary Information

**Additional file 1:** **Supplemental Figure S1. **Electroretinograms of patients and control. **Supplementary Table 1. **15 gene tubulointerstitial kidney disease panel (R202, PanelApp version 1.3). **Supplementary Table 2. **Retinal disorders panel (R32), PanelApp version 3.0). **Supplementary Table 3. **Additional alleles identified in patient 1 and patient 2.

## Data Availability

Data sharing is not applicable to this article as no datasets were generated or analysed during the current study.

## References

[1] Olinger E, Phakdeekitcharoen P, Caliskan Y, Orr S, Mabillard H, Pickles C, Tse Y, Wood K, Sayer JA (2022). Biallelic variants in TTC21B as a rare cause of early-onset arterial hypertension and tubuloglomerular kidney disease. Am J Med Genet C Semin Med Genet.

[2] Reisin, R., A. Perrin and P. García-Pavía. Time delays in the diagnosis and treatment of Fabry disease. Int J Clin Pract. 2017;71(1). 10.1111/ijcp.12914 28097762

[3] Schieppati A, Henter JI, Daina E, Aperia A (2008). Why rare diseases are an important medical and social issue. Lancet.

[4] ERA Registry: ERA Registry Annual Report 2020. Amsterdam: Amsterdam UMC, location AMC, Department of Medical Informatics; 2022.

[5] Kramer A, M Pippias, M Noordzij, VS Stel, N Afentakis, PM Ambühl, AM Andrusev, EA Fuster, FE Arribas Monzón, A Åsberg, M Barbullushi, M Bonthuis, FJ Caskey, P Castro de la Nuez, H Cernevskis, JM des Grottes, L Garneata, E Golan, MH Hemmelder, K Ioannou, F Jarraya, M Kolesnyk, K Komissarov, M Lassalle, F Macario, B Mahillo-Duran, AL Martín de Francisco, R Palsson, Ü Pechter, H Resic, B Rutkowski, C Santiuste de Pablos, N Seyahi, S Simic Ogrizovic, MF Slon Roblero, V Spustova, O Stojceva-Taneva, J Traynor, ZA Massy and KJ Jager. The European Renal Association - European Dialysis and Transplant Association (ERA-EDTA) Registry Annual Report 2015: a summary. Clin Kidney J. 2018;11(1):108-122 10.1093/ckj/sfx149 PMC5798130 29423210

[6] Saran R, Y Li, B Robinson, KC Abbott, LY Agodoa, J Ayanian, J Bragg-Gresham, R Balkrishnan, JL Chen, E Cope, PW Eggers, D Gillen, D Gipson, SM Hailpern, YN Hall, K He, W Herman, M Heung, RA Hirth, D Hutton, SJ Jacobsen, K Kalantar-Zadeh, CP Kovesdy, Y Lu, MZ Molnar, H Morgenstern, B Nallamothu, DV Nguyen, A M O’Hare, B Plattner, R Pisoni, FK Port, P Rao, CM Rhee, A Sakhuja, DE Schaubel, DT Selewski, V Shahinian, JJ Sim, P Song, E Streja, M Kurella Tamura, F Tentori, S White, K Woodside and RA Hirth. US Renal Data System 2015 annual data report: epidemiology of kidney disease in the United States. Am J Kidney Dis. 2016;67(3 Suppl 1):Svii, S1–305. 10.1053/j.ajkd.2015.12.014 PMC6643990 26925525

[7] Connaughton DM, Bukhari S, Conlon P, Cassidy E, O’Toole M, Mohamad M, Flanagan J, Butler T, O’Leary A, Wong L, O’Regan J, Moran S, O’Kelly P, Logan V, Griffin B, Griffin M, Lavin P, Little MA, Conlon P (2015). The Irish Kidney Gene Project–prevalence of family history in patients with kidney disease in Ireland. Nephron.

[8] Neild GH (2010). Primary renal disease in young adults with renal failure. Nephrol Dial Transplant.

[9] Titze S, Schmid M, Köttgen A, Busch M, Floege J, Wanner C, Kronenberg F, Eckardt KU (2015). Disease burden and risk profile in referred patients with moderate chronic kidney disease: composition of the German Chronic Kidney Disease (GCKD) cohort. Nephrol Dial Transplant.

[10] Genetics in chronic kidney disease: conclusions from a Kidney Disease: Improving Global Outcomes (KDIGO) controversies conference. Kidney Int. 2022;101(6):1126–1141. 10.1016/j.kint.2022.03.019 PMC9922534 35460632

[11] Knoers N, Antignac C, Bergmann C, Dahan K, Giglio S, Heidet L, Lipska-Ziętkiewicz BS, Noris M, Remuzzi G, Vargas-Poussou R, Schaefer F (2022). Genetic testing in the diagnosis of chronic kidney disease: recommendations for clinical practice. Nephrol Dial Transplant.

[12] Savige, J. Tips for testing adults with suspected genetic kidney disease. Am J Kidney Dis. 2023. 10.1053/j.ajkd.2023.10.011 38147894

[13] Snoek R, Stokman MF, Lichtenbelt KD, van Tilborg TC, Simcox CE, Paulussen ADC, Dreesen J, van Reekum F, Lely AT, Knoers N, de Die-Smulders CEM, van Eerde AM (2020). Preimplantation genetic testing for monogenic kidney disease. Clin J Am Soc Nephrol.

[14] Thompson, W. S., S. N. Babayev, M. L. McGowan, A. G. Kattah, M. J. Wick, E. M. Bendel-Stenzel, F. T. Chebib, P. C. Harris, N. K. Dahl, V. E. Torres and C. Hanna. State of the science and ethical considerations for preimplantation genetic testing for monogenic cystic kidney diseases and ciliopathies. J Am Soc Nephrol. 2023. 10.1681/ASN.0000000000000253 PMC10843344 37882743

[15] Smedley D, Smith KR, Martin A, Thomas EA, McDonagh EM, Cipriani V, Ellingford JM, Arno G, Tucci A, Vandrovcova J, Chan G, Williams HJ, Ratnaike T, Wei W, Stirrups K, Ibanez K, Moutsianas L, Wielscher M, Need A, Barnes MR, Vestito L, Buchanan J, Wordsworth S, Ashford S, Rehmström K, Li E, Fuller G, Twiss P, Spasic-Boskovic O, Halsall S, Floto RA, Poole K, Wagner A, Mehta SG, Gurnell M, Burrows N, James R, Penkett C, Dewhurst E, Gräf S, Mapeta R, Kasanicki M, Haworth A, Savage H, Babcock M, Reese MG, Bale M, Baple E, Boustred C, Brittain H, de Burca A, Bleda M, Devereau A, Halai D, Haraldsdottir E, Hyder Z, Kasperaviciute D, Patch C, Polychronopoulos D, Matchan A, Sultana R, Ryten M, Tavares ALT, Tregidgo C, Turnbull C, Welland M, Wood S, Snow C, Williams E, Leigh S, Foulger RE, Daugherty LC, Niblock O, Leong IUS, Wright CF, Davies J, Crichton C, Welch J, Woods K, Abulhoul L, Aurora P, Bockenhauer D, Broomfield A, Cleary MA, Lam T, Dattani M, Footitt E, Ganesan V, Grunewald S, Compeyrot-Lacassagne S, Muntoni F, Pilkington C, Quinlivan R, Thapar N, Wallis C, Wedderburn LR, Worth A, Bueser T, Compton C, Deshpande C, Fassihi H, Haque E, Izatt L, Josifova D, Mohammed S, Robert L, Rose S, Ruddy D, Sarkany R, Say G, Shaw AC, Wolejko A, Habib B, Burns G, Hunter S, Grocock RJ, Humphray SJ, Robinson PN, Haendel M, Simpson MA, Banka S, Clayton-Smith J, Douzgou S, Hall G, Thomas HB, O’Keefe RT, Michaelides M, Moore AT, Malka S, Pontikos N, Browning AC, Straub V, Gorman GS, Horvath R, Quinton R, Schaefer AM, Yu-Wai-Man P, Turnbull DM, McFarland R, Taylor RW, O’Connor E, Yip J, Newland K, Morris HR, Polke J, Wood NW, Campbell C, Camps C, Gibson K, Koelling N, Lester T, Németh AH, Palles C, Patel S, Roy NBA, Sen A, Taylor J, Cacheiro P, Jacobsen JO, Seaby EG, Davison V, Chitty L, Douglas A, Naresh K, McMullan D, Ellard S, Temple IK, Mumford AD, Wilson G, Beales P, Bitner-Glindzicz M, Black G, Bradley JR, Brennan P, Burn J, Chinnery PF, Elliott P, Flinter F, Houlden H, Irving M, Newman W, Rahman S, Sayer JA, Taylor JC, Webster AR, Wilkie AOM, Ouwehand WH, Raymond FL, Chisholm J, Hill S, Bentley D, Scott RH, Fowler T, Rendon A, Caulfield M (2021). 100,000 genomes pilot on rare-disease diagnosis in health care - preliminary report. N Engl J Med.

[16] Groopman EE, Marasa M, Cameron-Christie S, Petrovski S, Aggarwal VS, Milo-Rasouly H, Li Y, Zhang J, Nestor J, Krithivasan P, Lam WY, Mitrotti A, Piva S, Kil BH, Chatterjee D, Reingold R, Bradbury D, DiVecchia M, Snyder H, Mu X, Mehl K, Balderes O, Fasel DA, Weng C, Radhakrishnan J, Canetta P, Appel GB, Bomback AS, Ahn W, Uy NS, Alam S, Cohen DJ, Crew RJ, Dube GK, Rao MK, Kamalakaran S, Copeland B, Ren Z, Bridgers J, Malone CD, Mebane CM, Dagaonkar N, Fellström BC, Haefliger C, Mohan S, Sanna-Cherchi S, Kiryluk K, Fleckner J, March R, Platt A, Goldstein DB, Gharavi AG (2019). Diagnostic utility of exome sequencing for kidney disease. N Engl J Med.

[17] Dahl NK, MS Bloom, FT Chebib, D Clark, M Westemeyer, S Jandeska, Z Zhang, H Milo-Rasouly, V Kolupaeva, M Marasa, V Broumand, RA Fatica, DS Raj, ZP Demko, K Marshall, S Punj, H Tabriziani, S Bhorade and AG Gharavi. The clinical utility of genetic testing in the diagnosis and management of adults with chronic kidney disease. J Am Soc Nephrol. 2023. 10.1681/ASN.0000000000000249 PMC10703084 37794564

[18] Millions of people with rare diseases to benefit from faster diagnosis and better access to treatment. 2022. Retrieved 31/01/2023, 2023, from https://www.gov.uk/government/news/millions-of-people-with-rare-diseases-to-benefit-from-faster-diagnosis-and-better-access-to-treatment.

[19] Lemoine H, Raud L, Foulquier F, Sayer JA, Lambert B, Olinger E, Lefèvre S, Knebelmann B, Harris PC, Trouvé P, Desprès A, Duneau G, Matignon M, Poyet A, Jourde-Chiche N, Guerrot D, Lemoine S, Seret G, Barroso-Gil M, Bingham C, Gilbert R, Le Meur Y, Audrézet MP, Cornec-Le Gall E (2022). Monoallelic pathogenic ALG5 variants cause atypical polycystic kidney disease and interstitial fibrosis. Am J Hum Genet.

[20] Senum SR, Li YSM, Benson KA, Joli G, Olinger E, Lavu S, Madsen CD, Gregory AV, Neatu R, Kline TL, Audrézet MP, Outeda P, Nau CB, Meijer E, Ali H, Steinman TI, Mrug M, Phelan PJ, Watnick TJ, Peters DJM, Ong ACM, Conlon PJ, Perrone RD, Cornec-Le Gall E, Hogan MC, Torres VE, Sayer JA, Harris PC (2022). Monoallelic IFT140 pathogenic variants are an important cause of the autosomal dominant polycystic kidney-spectrum phenotype. Am J Hum Genet.

[21] Gast C, Pengelly RJ, Lyon M, Bunyan DJ, Seaby EG, Graham N, Venkat-Raman G, Ennis S (2016). Collagen (COL4A) mutations are the most frequent mutations underlying adult focal segmental glomerulosclerosis. Nephrol Dial Transplant.

[22] Eckardt KU, Alper SL, Antignac C, Bleyer AJ, Chauveau D, Dahan K, Deltas C, Hosking A, Kmoch S, Rampoldi L, Wiesener M, Wolf MT, Devuyst O (2015). Autosomal dominant tubulointerstitial kidney disease: diagnosis, classification, and management–a KDIGO consensus report. Kidney Int.

[23] Sayer, J. A. Nephronophthisis and medullary cystic kidney disease: overview. Oxford Textbook of Clinical Nephrology: Three-Volume Pack. N. Turner, N. N. Turner, N. N. Turner et al., Oxford University Press: 0. 2015.

[24] Srivastava S, Sayer JA (2014). Nephronophthisis. J Pediatr Genet.

[25] Georges B, Cosyns JP, Dahan K, Snyers B, Carlier B, Loute G, Pirson Y (2000). Late-onset renal failure in Senior-Loken syndrome. Am J Kidney Dis.

[26] Levy M, Feingold J (2000). Estimating prevalence in single-gene kidney diseases progressing to renal failure. Kidney Int.

[27] Haghighi A, Savaj S, Haghighi-Kakhki H, Benoit V, Grisart B, Dahan K (2016). Identification of an NPHP1 deletion causing adult form of nephronophthisis. Ir J Med Sci.

[28] Hoefele J, Nayir A, Chaki M, Imm A, Allen SJ, Otto EA, Hildebrandt F (2011). Pseudodominant inheritance of nephronophthisis caused by a homozygous NPHP1 deletion. Pediatr Nephrol.

[29] Ala-Mello S, Koskimies O, Rapola J, Kääriäinen H (1999). Nephronophthisis in Finland: epidemiology and comparison of genetically classified subgroups. Eur J Hum Genet.

[30] Simms RJ, Eley L, Sayer JA (2009). Nephronophthisis. Eur J Hum Genet.

[31] Zollinger HU, MJ Mihatsch, A Edefonti, F Gaboardi, E Imbasciati and T Lennert. Nephronophthisis (medullary cystic disease of the kidney). A study using electron microscopy, immunofluorescence, and a review of the morphological findings. Helv Paediatr Acta. 1980;35(6):509–530. 7009503

[32] Snoek R, van Setten J, Keating BJ, Israni AK, Jacobson PA, Oetting WS, Matas AJ, Mannon RB, Zhang Z, Zhang W, Hao K, Murphy B, Reindl-Schwaighofer R, Heinzl A, Oberbauer R, Viklicky O, Conlon PJ, Stapleton CP, Bakker SJL, Snieder H, Peters EDJ, van der Zwaag B, Knoers N, de Borst MH, van Eerde AM (2018). NPHP1 (nephrocystin-1) gene deletions cause adult-onset ESRD. J Am Soc Nephrol.

[33] Loken AC, Hanssen O, Halvorsen S, Jolster NJ (1961). Hereditary renal dysplasia and blindness. Acta Paediatr (Stockh).

[34] Senior B, Friedmann AI, Braudo JL (1961). Juvenile familial nephropathy with tapetoretinal degeneration. A new oculorenal dystrophy. Am J Ophthalmol.

[35] Caridi G, Murer L, Bellantuono R, Sorino P, Caringella DA, Gusmano R, Ghiggeri GM (1998). Renal-retinal syndromes: association of retinal anomalies and recessive nephronophthisis in patients with homozygous deletion of the NPH1 locus. Am J Kidney Dis.

[36] Birtel J, Spital G, Book M, Habbig S, Bäumner S, Riehmer V, Beck BB, Rosenkranz D, Bolz HJ, Dahmer-Heath M, Herrmann P, König J, Charbel Issa P (2021). NPHP1 gene-associated nephronophthisis is associated with an occult retinopathy. Kidney Int.

[37] Coppieters F, Casteels I, Meire F, De Jaegere S, Hooghe S, van Regemorter N, Van Esch H, Matuleviciene A, Nunes L, Meersschaut V, Walraedt S, Standaert L, Coucke P, Hoeben H, Kroes HY, Vande Walle J, de Ravel T, Leroy BP, De Baere E (2010). Genetic screening of LCA in Belgium: predominance of CEP290 and identification of potential modifier alleles in AHI1 of CEP290-related phenotypes. Hum Mutat.

[38] Sayer JA, Otto EA, O’Toole JF, Nurnberg G, Kennedy MA, Becker C, Hennies HC, Helou J, Attanasio M, Fausett BV, Utsch B, Khanna H, Liu Y, Drummond I, Kawakami I, Kusakabe T, Tsuda M, Ma L, Lee H, Larson RG, Allen SJ, Wilkinson CJ, Nigg EA, Shou C, Lillo C, Williams DS, Hoppe B, Kemper MJ, Neuhaus T, Parisi MA, Glass IA, Petry M, Kispert A, Gloy J, Ganner A, Walz G, Zhu X, Goldman D, Nurnberg P, Swaroop A, Leroux MR, Hildebrandt F (2006). The centrosomal protein nephrocystin-6 is mutated in Joubert syndrome and activates transcription factor ATF4. Nat Genet.

[39] Sherer DW, Bercovitch L, Lebwohl M (2001). Pseudoxanthoma elasticum: significance of limited phenotypic expression in parents of affected offspring. J Am Acad Dermatol.

[40] Reiners J, van Wijk E, Märker T, Zimmermann U, Jürgens K, te Brinke H, Overlack N, Roepman R, Knipper M, Kremer H, Wolfrum U (2005). Scaffold protein harmonin (USH1C) provides molecular links between Usher syndrome type 1 and type 2. Hum Mol Genet.

[41] Möller CG, Kimberling WJ, Davenport SL, Priluck I, White V, Biscone-Halterman K, Odkvist LM, Brookhouser PE, Lund G, Grissom TJ (1989). Usher syndrome: an otoneurologic study. Laryngoscope.

[42] Pinckers A, van Aarem A, Brink H (1994). The electrooculogram in heterozygote carriers of Usher syndrome, retinitis pigmentosa, neuronal ceroid lipofuscinosis, senior syndrome and choroideremia. Ophthalmic Genet.

[43] Mann N, Braun DA, Amann K, Tan W, Shril S, Connaughton DM, Nakayama M, Schneider R, Kitzler TM, van der Ven AT, Chen J, Ityel H, Vivante A, Majmundar AJ, Daga A, Warejko JK, Lovric S, Ashraf S, Jobst-Schwan T, Widmeier E, Hugo H, Mane SM, Spaneas L, Somers MJG, Ferguson MA, Traum AZ, Stein DR, Baum MA, Daouk GH, Lifton RP, Manzi S, Vakili K, Kim HB, Rodig NM, Hildebrandt F (2019). Whole-exome sequencing enables a precision medicine approach for kidney transplant recipients. J Am Soc Nephrol.

[44] Prakash S, Gharavi AG (2015). Diagnosing kidney disease in the genetic era. Curr Opin Nephrol Hypertens.

[45] Stone HK, VandenHeuvel K, Bondoc A, Flores FX, Hooper DK, Varnell CD (2021). Primary hyperoxaluria diagnosed after kidney transplant: a review of the literature and case report of aggressive renal replacement therapy and lumasiran to prevent allograft loss. Am J Transplant.

[46] Ashraf S, Gee HY, Woerner S, Xie LX, Vega-Warner V, Lovric S, Fang H, Song X, Cattran DC, Avila-Casado C, Paterson AD, Nitschké P, Bole-Feysot C, Cochat P, Esteve-Rudd J, Haberberger B, Allen SJ, Zhou W, Airik R, Otto EA, Barua M, Al-Hamed MH, Kari JA, Evans J, Bierzynska A, Saleem MA, Böckenhauer D, Kleta R, El Desoky S, Hacihamdioglu DO, Gok F, Washburn J, Wiggins RC, Choi M, Lifton RP, Levy S, Han Z, Salviati L, Prokisch H, Williams DS, Pollak M, Clarke CF, Pei Y, Antignac C, Hildebrandt F (2013). ADCK4 mutations promote steroid-resistant nephrotic syndrome through CoQ10 biosynthesis disruption. J Clin Invest.

[47] Heeringa SF, Chernin G, Chaki M, Zhou W, Sloan AJ, Ji Z, Xie LX, Salviati L, Hurd TW, Vega-Warner V, Killen PD, Raphael Y, Ashraf S, Ovunc B, Schoeb DS, McLaughlin HM, Airik R, Vlangos CN, Gbadegesin R, Hinkes B, Saisawat P, Trevisson E, Doimo M, Casarin A, Pertegato V, Giorgi G, Prokisch H, Rötig A, Nürnberg G, Becker C, Wang S, Ozaltin F, Topaloglu R, Bakkaloglu A, Bakkaloglu SA, Müller D, Beissert A, Mir S, Berdeli A, Varpizen S, Zenker M, Matejas V, Santos-Ocaña C, Navas P, Kusakabe T, Kispert A, Akman S, Soliman NA, Krick S, Mundel P, Reiser J, Nürnberg P, Clarke CF, Wiggins RC, Faul C, Hildebrandt F (2011). COQ6 mutations in human patients produce nephrotic syndrome with sensorineural deafness. J Clin Invest.

[48] Montini G, Malaventura C, Salviati L (2008). Early coenzyme Q10 supplementation in primary coenzyme Q10 deficiency. N Engl J Med.

[49] Ozaltin F (2014). Primary coenzyme Q10 (CoQ 10) deficiencies and related nephropathies. Pediatr Nephrol.

[50] Mabillard H, Olinger E, Sayer JA (2022). UMOD and you! Explaining a rare disease diagnosis. J Rare Dis (Berlin).

[51] van Eerde AM, Krediet CT, Rookmaaker MB, van Reekum FE, Knoers NV, Lely AT (2016). Pre-pregnancy advice in chronic kidney disease: do not forget genetic counseling. Kidney Int.

