# Supplementary material for: Diverse retinal-kidney phenotypes associated with NPHP1 homozygous whole-gene deletions in patients with kidney failure
Source: J Rare Dis (Berlin). 2024 Mar 1;3(1):7. doi: 10.1007/s44162-024-00031-4 (PMC10904492; doi:10.1007/s44162-024-00031-4)
Supplement: Supplementary file 1 — Additional file 1: Supplemental Figure S1. Electroretinograms of patients and control. Supplementary Table 1. 15 gene tubulointerstitial kidney disease panel (R202, PanelApp version 1.3). Supplementary Table 2. Retinal disorders panel (R32), PanelApp version 3.0). Supplementary Table 3. Additional alleles identified in patient 1 and patient 2. [file 44162_2024_31_MOESM1_ESM.docx]

**Supplementary Materials**

**Diverse retinal-kidney phenotypes associated with *NPHP1* homozygous whole gene deletions in patients with kidney failure**

Gavin Esson^1^, Ian Logan^1^, Katrina Wood^2^ Andrew C. Browning^3^ and John A. Sayer^1,4,5^


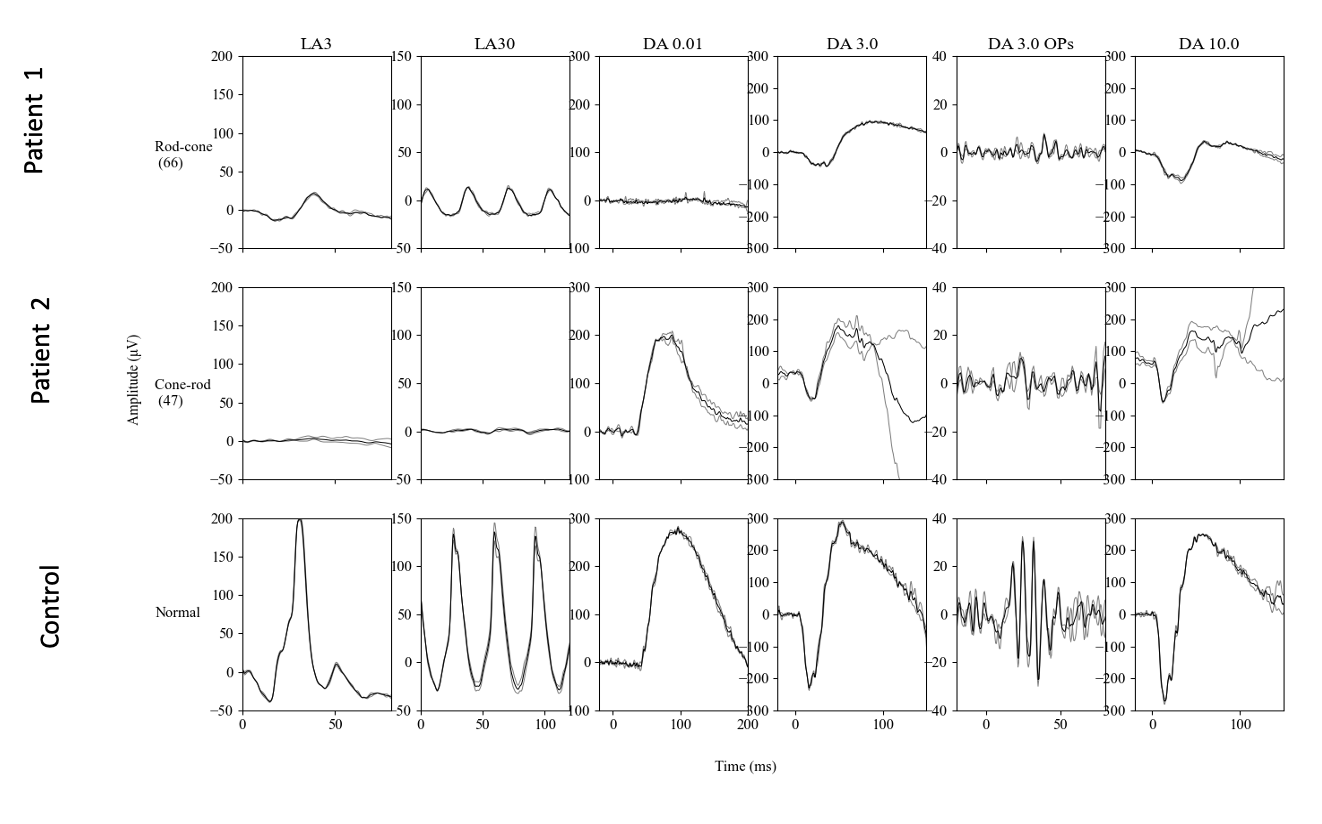


**Supplemental Figure S1. Electroretinograms of patients and control**

International Society for Clinical Electrophysiology of Vision (ISCEV) standard full filed electroretinogram (ERG) of patient 1 (upper traces) and patient 2 (middle traces). The lower traces are from a normal age matched control. The patient 1 traces demonstrate barely detectable rod photoreceptor function (DA 0.01) and attenuated cone function (LA 3.0 and LA 30 Hz) consistent with a rod/cone dystrophy. The patient 2 traces demonstrate barely detectable cone function (LA 3.0 and LA 30 Hz) while rod photoreceptor function (DA 0.01) is well preserved, consistent with a cone dystrophy.

**Supplementary Table 1. 15 gene tubulointerstitial kidney disease panel (R202, PanelApp version 1.3)**

| *ANKS6* |
| --- |
| *CEP164* |
| *CEP83* |
| *GATM* |
| *HNF1B* |
| *INVS* |
| *MAPKBP1* |
| *NPHP1* |
| *NPHP3* |
| *NPHP4* |
| *REN* |
| *TMEM67* |
| *TTC21B* |
| *UMOD* |
| *WDR19* |

**Supplementary Table 2. Retinal disorders panel (R32), PanelApp version 3.0)**

| ABCA4 | C21orf2 | CROCC | GNPTG | LRAT | OPN1LW | PRTFDC1 | SPTLC1 | WDR19 |
| --- | --- | --- | --- | --- | --- | --- | --- | --- |
| ABCC6 | C2orf71 | CRX | GP1BA | LRIT3 | OPN1MW | RAB28 | SPTLC2 | WFS1 |
| ABHD12 | C3 | CSPP1 | GPR143 | LRMDA | OPN1SW | RAX2 | SRD5A3 | WHRN |
| ACBD5 | C5orf42 | CTC1 | GPR179 | LRP1 | OR2M7 | RB1 | SSBP1 | WT1 |
| ACO2 | C8orf37 | CTNNA1 | GRIP1 | LRP2 | OTX2 | RBP3 | STRA6 | ZFYVE26 |
| ADAM9 | CA4 | CTNNB1 | GRK1 | LRP5 | P3H2 | RBP4 | TCTN1 | ZNF408 |
| ADAMTS18 | CABP4 | CTSD | GRM6 | LZTFL1 | PAK2 | RCBTB1 | TCTN2 | ZNF423 |
| ADGRA3 | CACNA1F | CTSF | GRN | MAK | PANK2 | RD3 | TCTN3 | ZNF513 |
| ADGRV1 | CACNA2D4 | CUBN | GUCA1A | MAPKAPK3 | PAX2 | RDH11 | TEAD1 | ZPR1 |
| ADIPOR1 | CAPN5 | CWC27 | GUCA1B | MED12 | PAX6 | RDH12 | TEX28 |  |
| AFG3L2 | CC2D2A | CYP1B1 | GUCY2D | MERTK | PCDH15 | RDH5 | TIMM8A |  |
| AGBL5 | CCT2 | CYP27A1 | HARS | MFN2 | PCYT1A | REEP6 | TIMP3 |  |
| AHI1 | CCZ1B | CYP2R1 | HCCS | MFRP | PDAP1 | RGR | TINF2 |  |
| AHR | CDH23 | CYP4V2 | HGSNAT | MFSD8 | PDE6A | RGS9 | TMEM126A |  |
| AIPL1 | CDH3 | DHDDS | HK1 | MIR204 | PDE6B | RGS9BP | TMEM216 |  |
| AIRE | CDHR1 | DHX38 | HKDC1 | MKKS | PDE6C | RHO | TMEM218 |  |
| ALDH3A2 | CEP164 | DMD | HMCN1 | MKS1 | PDE6G | RIMS1 | TMEM231 |  |
| ALMS1 | CEP19 | DRAM2 | HMX1 | MMACHC | PDE6H | RIMS2 | TMEM237 |  |
| ALPK1 | CEP250 | DTHD1 | HTRA1 | MSTO1 | PDSS1 | RLBP1 | TMEM67 |  |
| AMACR | CEP290 | DYNC2H1 | IDH3A | MT-ATP6 | PDZD7 | RNU4ATAC | TOPORS |  |
| AMN | CEP41 | EFEMP1 | IDH3B | MT-ND1 | PEX1 | ROM1 | TPP1 |  |
| AP3B2 | CEP78 | ELOVL1 | IFT140 | MT-ND4 | PEX2 | RP1 | TRAF3IP1 |  |
| ARHGEF18 | CERKL | ELOVL4 | IFT172 | MT-ND6 | PEX6 | RP1L1 | TREX1 |  |
| ARL13B | CFB | EMC1 | IFT27 | MT-TH | PEX7 | RP2 | TRIM32 |  |
| ARL2BP | CFH | ERCC6 | IFT74 | MT-TL1 | PGK1 | RP9 | TRNT1 |  |
| ARL3 | CHM | ERCC8 | IFT81 | MTTP | PHYH | RPE65 | TRPM1 |  |
| ARL6 | CIB2 | ESPN | IKBKG | MT-TP | PITPNM3 | RPGR | TSPAN12 |  |
| ARMS2 | CLCC1 | EVR3 | IMPDH1 | MT-TS2 | PITX2 | RPGRIP1 | TTC21B |  |
| ARSG | CLN3 | EXOSC2 | IMPG1 | MVK | PITX3 | RPGRIP1L | TTC8 |  |
| ASRGL1 | CLN5 | EYS | IMPG2 | MYO7A | PLA2G5 | RS1 | TTLL5 |  |
| ATF6 | CLN6 | FAM161A | INPP5E | MYOC | PLD4 | RTN4IP1 | TTPA |  |
| ATOH7 | CLN8 | FAM57B | INVS | NAALADL1 | PLK4 | SAG | TUB |  |
| ATP13A2 | CLRN1 | FAM71A | IQCB1 | NBAS | PNPLA6 | SAMD11 | TUBB4B |  |
| ATXN7 | CLUAP1 | FBLN5 | IRX5 | NDP | POC1B | SCAPER | TUBGCP4 |  |
| B3GLCT | CNGA1 | FLVCR1 | IRX6 | NEK2 | POC5 | SDCCAG8 | TUBGCP6 |  |
| BBIP1 | CNGA3 | FOXC1 | ITIH2 | NEUROD1 | PODNL1 | SEMA4A | TULP1 |  |
| BBS1 | CNGB1 | FOXE3 | ITM2B | NMNAT1 | POMGNT1 | SLC24A1 | TYR |  |
| BBS10 | CNGB3 | FOXI2 | JAG1 | NPHP1 | POMT1 | SLC24A5 | TYRP1 |  |
| BBS12 | CNNM4 | FRAS1 | KCNJ13 | NPHP3 | POMZP3 | SLC25A46 | UBAP1L |  |
| BBS2 | COL11A1 | FREM1 | KCNV2 | NPHP4 | PPT1 | SLC37A3 | UNC119 |  |
| BBS4 | COL11A2 | FREM2 | KCTD7 | NR2E3 | PRCD | SLC38A8 | USH1C |  |
| BBS5 | COL18A1 | FRMD7 | KIAA1549 | NR2F1 | PRDM13 | SLC45A2 | USH1G |  |
| BBS7 | COL2A1 | FSCN2 | KIF11 | NRL | PROM1 | SLC6A6 | USH2A |  |
| BBS9 | COL4A1 | FUT5 | KIF3B | NUMB | PRPF3 | SLC7A14 | USP45 |  |
| BCOR | COL9A1 | FZD4 | KIF7 | NYX | PRPF31 | SMOC1 | VAX1 |  |
| BEST1 | COL9A2 | GDF6 | KIZ | OAT | PRPF4 | SNRNP200 | VCAN |  |
| BMP4 | COQ2 | GNAT1 | KLHL7 | OCA2 | PRPF6 | SOX2 | VPS13B |  |
| C12orf65 | COQ4 | GNAT2 | LAMA1 | OFD1 | PRPF8 | SPATA7 | VSX2 |  |
| C1QTNF5 | COQ5 | GNB3 | LCA5 | OPA1 | PRPH2 | SPG7 | WASF3 |  |
| C2 | CRB1 | GNPTAB | LIG3 | OPA3 | PRPS1 | SPP2 | WDPCP |  |

**Supplementary Table 3. Additional alleles identified in patient 1 and patient 2**

| **Patient 1** | | | | | |
| --- | --- | --- | --- | --- | --- |
| Gene | Zygosity | HGVS description | Location | Classification | Allele Frequency (gnomAD v.4.0.0) |
| *NPHP1* | Homozygous | NM_000272.4: (*NPHP1*) whole gene deletion | Chr2 (GRCh38) g.(?_110962545)_(110881368_?)x0 | Pathogenic  (PM2_Moderate; PM3_Very Strong; PVS1_Very Strong) | 0.002198 |
| *ABCC6* | Heterozygous | NM_001171.5 (*ABCC6*); c.3421C>T; p.(Arg1141Ter) | Chr16 (GRCh38):g.(1613078)G>A | Pathogenic  (PM2_Moderate; PVS1_Very Strong; PM3_Very Strong) | 0.001613 |

| **Patient 2** | | | | | |
| --- | --- | --- | --- | --- | --- |
| Gene | Zygosity | HGVS description | Location | Variant Classification* | Allele Frequency (gnomAD v.4.0.0) |
| *NPHP1* | Homozygous | NM_000272.4: (*NPHP1*) whole gene deletion | Chr2 (GRCh38): g.(110095625-110226966del) | Pathogenic  (PM2_Moderate; PM3_Very Strong; PVS1_Very Strong) | 0.002198 |
| *USH1C* | Heterozygous | NM_153676.4 (*USH1C*);  c.586C>T; p.(Arg196Ter) | Chr11 (GRCh38): g.(17547982G>A) | Pathogenic | 0.000008895 |

*Variant classification using ACMG guidelines is given.
